# Longitudinal Walking Analysis in Hemiparetic Patients Using Wearable Motion Sensors: Is There Convergence Between Body Sides?

**DOI:** 10.3389/fbioe.2018.00057

**Published:** 2018-05-31

**Authors:** Adrian Derungs, Corina Schuster-Amft, Oliver Amft

**Affiliations:** ^1^Chair of eHealth and mHealth, Friedrich-Alexander Universität Erlangen-Nürnberg, Erlangen, Germany; ^2^Research Department, Reha Rheinfelden, Rheinfelden, Switzerland; ^3^Institute for Rehabilitation and Performance Technology, Bern University of Applied Sciences, Burgdorf, Switzerland; ^4^Department of Sport, Exericse and Health, University of Basel, Basel, Switzerland

**Keywords:** free-living, inertial measurement units, stroke, rehabilitation, trend, unsupervised

## Abstract

**Background:** Longitudinal movement parameter analysis of hemiparetic patients over several months could reveal potential recovery trends and help clinicians adapting therapy strategies to maximize recovery outcome. Wearable sensors offer potential for day-long movement recordings in realistic rehabilitation settings including activities of daily living, e.g., walking. The measurement of walking-related movement parameters of affected and non-affected body sides are of interest to determine mobility and investigate recovery trends.

**Methods:** By comparing movement of both body sides, recovery trends across the rehabilitation duration were investigated. We derived and validated selected walking segments from free-living, day-long movement by using rules that do not require data-based training or data annotations. Automatic stride segmentation using peak detection was applied to walking segments. Movement parameters during walking were extracted, including stride count, stride duration, cadence, and sway. Finally, linear regression models over each movement parameter were derived to forecast the moment of convergence between body sides. Convergence points were expressed as duration and investigated in a patient observation study.

**Results:** Convergence was analyzed in walking-related movement parameters in an outpatient study including totally 102 full-day recordings of inertial movement data from 11 hemiparetic patients. The recordings were performed over several months in a day-care centre. Validation of the walking extraction method from sensor data yielded sensitivities up to 80 % and specificity above 94 % on average. Comparison of automatically and manually derived movement parameters showed average relative errors below 6 % between affected and non-affected body sides. Movement parameter variability within and across patients was observed and confirmed by case reports, reflecting individual patient behavior.

**Conclusion:** Convergence points were proposed as intuitive metric, which could facilitate training personalization for patients according to their individual needs. Our continuous movement parameter extraction and analysis, was feasible for realistic, day-long recordings without annotations. Visualizations of movement parameter trends and convergence points indicated that individual habits and patient therapies were reflected in walking and mobility. Context information of clinical case reports supported trend and convergence interpretation. Inconsistent convergence point estimation suggested individually varying deficiencies. Long-term recovery monitoring using convergence points could support patient-specific training strategies in future remote rehabilitation.

## Introduction

Analyzing movement parameters in patients with a hemiparesis over weeks and months could help clinicians to understand behavioral changes and reveal potential recovery trends beside classical clinical assessments. Wearable motion sensors can continuously assess affected and non-affected body sides during an extended stroke recovery process and could therefore enable rehabilitation experts to plan goal setting and therapy adaptations. Continuous measurement and analysis may facilitate devising personalized therapy and maximize recovery outcome. The importance of unsupervised movement parameter analysis over weeks and months was emphasized by Patterson et al. (2010) and further discussed by Marschollek et al. (2012), highlighting general benefits in health-care using wearable sensors due to “long-term, objective measurement under daily-life, unsupervised conditions.” However, to date, wearable sensors and automatic movement parameter analysis for free-living trend analysis in patients after stroke received limited attention.

Research has shown that movement-related features derived using wearable inertial measurement sensor units (IMUs), including 3-axis accelerometers, gyroscopes, and magnetic field sensors, can be used to estimate clinical scores according to clinically supervised assessments e.g., the Wolf Motor Function Test (WMFT) (Wade et al., 2010), the Fugl-Meyer-Assessment (FMA) (Del Din et al., 2011) or the National Institute of Health Stroke Score (NIHSS) motor index (Gubbi et al., 2013). Clinical assessments, that include wearable sensors, focus on selected, specifically designed, and isolated motor function tasks, where patients are specifically asked to perform certain tasks (reaching, grasping, and similar) in controlled lab-like settings, guided and assessed by clinicians. Typically, the tool-chain to analyse wearable sensor data involved pattern classification (Parnandi et al., 2010; Patel et al., 2010) and regression (Hester et al., 2006; Del Din et al., 2011) techniques, to derive objective, quantitative measurements, and estimates of clinical scores. While, clinical assessments and their corresponding scores may be replicated with estimations using wearable sensors, mainly short-term measurements derived in lab-controlled settings were investigated. Especially in the rehabilitation of hemiparetic patients, e.g., after stroke, where recovery can be a gradual process over weeks, months, and even years, continuous measurement and quantification are needed.

Various sensor technologies have been used and motion sensors including accelerometers (Moe-Nilssen and Helbostad, 2004; Zijlstra, 2004; Senden et al., 2009; Sant'Anna and Wickström, 2010) or gyroscopes (Greene et al., 2010; Abaid et al., 2013; Fraccaro et al., 2014) were frequently described for gait and movement analysis. Parisi et al. (2016) applied a Shimmer3 IMU, attached to the lower trunk, for movement analysis in stroke patients. Results of their investigation showed high correlation (Pearson, *r* ≥ 0.82) between spatio-temporal features derived from the IMU and an optical reference system. Beside motion sensors, stationary optical systems (Whittle, 1996; Boutaayamou et al., 2015) and pressure sensor systems (Chen et al., 2005; Sant'Anna and Wickström, 2010) were used to investigate gait cycles. Wearable sensors were also deployed to analyse movement parameters in patients suffering from Parkinson's disease (PD) (Hundza et al., 2014), cerebral palsy (Strohrmann et al., 2013), impacts of surgical interventions, e.g., hip arthroplasty (Aminian et al., 1999) or for behavior analysis of the elderly (Hollman et al., 2011; Fraccaro et al., 2014). Often sensor technology for movement analysis studies were tailored to derive and investigate gait cycles in controlled clinical settings, e.g., using a treadmill (Abaid et al., 2013; Evans and Arvind, 2014), where patients followed instructions given by clinicians. These controlled settings allowed experts to take annotations, but resulted in limited amount of sensor data due to time and cost required. Generalization to realistic daily life settings remain unclear (Salarian et al., 2004; Chen et al., 2005). In particular, solutions for transferring lab-controlled analysis approaches to free-living are required to monitor recovery trends over multiple weeks and months. Algorithms to derive movement parameters including stride length, velocity or distance, typically include peak detection approaches (Aminian et al., 1999; Greene et al., 2010; Sant'Anna and Wickström, 2010; Hundza et al., 2014) and involve determining thresholds, either empirically or using experts knowledge. An extensive discussion on sensors and gait segmentation was provided by Taborri et al. (2016). In contrast, in this work we present a longitudinal observation study in a day-care centre, and investigate unsupervised approaches for mobility analysis, suitable for free-living.

Rehabilitation strategies, particularly those investigated with patients after stroke, emphasize the importance of walking as predictor for rehabilitation outcome and describe walking as major rehabilitation goal, to regain mobility and subsequently increased independence (Olney and Richards, 1996; Gordon, 2004; Duncan et al., 2005). Although concepts of life-long learning were controversially discussed, Dobkin argued that continuous (walking)-progress in patients after stroke is realistic (Dobkin, 2004). Longitudinal studies, using wearable sensors, may help to understand the life-long learning of patients with a hemiparesis by objective measurements of behavioral changes and recovery trends. Faralli et al. (2013), and Takeuchi and Izumi (2013) further emphasized the importance of a comprehensive and intensive rehabilitation to regain or improve motor function by different processes, i.e., neurogenesis (new neuron production) and plasticity (reorganization). Further, it has been shown that realistic rehabilitation, including activities of daily living (ADL) and task-specific practice, e.g., described in the Extended Barthel Index (EBI) assessment, were beneficial to support and induce recovery (Winstein et al., 2016). Movement quality could be evaluated in daily life using activity monitoring, indicating when patients were active as shown by van Meulen et al. (2016). However only 201 min of data derived from two patients were analyzed. So far, longitudinal studies analyzing recovery trends in hemiparetic outpatients during extended rehabilitation periods in a day-care centre were not investigated.

We analyse walking movement and mobility behavior in free-living, regarding inter- and intra-patient differences, and investigate whether recovery trends in patients with a hemiparesis during a multi-week outpatient rehabilitation period could be interpreted. In particular, this paper provides the following contributions:

1. We evaluate walking-related movement parameters of 11 outpatients after stroke or brain tumor extraction from day-long recordings derived in a free-living rehabilitation setting of a rehabilitation day-care centre. A total of 102 recording days were acquired over several months. We show that wearable motion sensors capture walking characteristics related to patients' individual behavior, therapy schedules, and health conditions.
2. We present a longitudinal movement parameter study and analysis during walking using wearable motion sensors. We extract walking segments, determine strides, and compare affected and non-affected body sides to describe movement changes. In the present work, we highlight the bilateral trend analysis and discuss potential for free-living analysis. We detail our analysis for three typical patients using clinical case reports.
3. We investigate potential recovery trends in hemiparetic patients by comparing body sides and propose a new, regression-based approach to quantify movement parameters using convergence points.

## Methods

We first detail our evaluation study followed by the description of the bilateral trend analysis.

### Evaluation study

#### Participants

We included 11 patients with a hemiparesis in our study (5 females, aged 34–75 years, 4 wheelchair users). Inclusion criteria were: stroke or brain tumor extraction with subsequent upper and/or lower motor function deficits, including wheelchair reliant patients. The inclusion of wheelchair users was of interest to demonstrate patients' continuity and potential recovery trends using wearable sensors. Due to the longitudinal study design, we expected to observe changes in walking behavior of initially wheelchair-dependent patients toward independent walking. Patients were excluded if presenting additional motor function impairments caused by neurological diseases. Walking aids, e.g., sticks or foot orthosis, were not an exclusion criteria. Overall, eight patients after stroke and three patients after brain tumor extraction were included in the study. Study participants visited the day-care centre at the rehabilitation clinic Reha Rheinfelden in Switzerland. All patients signed a written consent form for study participation and publication of results before data recording began. The study was approved by the Swiss cantonal Ethics committee of the canton Aargau, Switzerland (Application number: 2013/009). During the data recording period from December 2013 to May 2014, patients spent between 16 to 79 days at the day-care centre. Days after stroke or brain tumor extraction spanned from 48 to 335 days. Patients' details, including EBI-scores, are summarized in Table 1.

**Table 1:** Patient information.

**ID**	**Cause of impairment**	**Locomotion (type)**	**Gender**	**Affected (side)**	**Age (years)**	**Rehab (days)**	**Rec. (days)**	**DAS (days)**	**EBI^⋆^ compl**	**EBI Δ_compl_**	**EBI^⋆^ walk**	**EBI Δ_walk_**
1	Stroke	Wheelchair	M	Left	57	79	11	335	51	+8	1	+1
2	Stroke	Walk	M	Right	47	18	8	135	63	+1	4	0
3	Stroke	Wheelchair	M	Right	53	77	10	164	51	+10	0	0
4	Stroke	Walk	F	Left	52	16	7	295	60	+1	4	0
5	Stroke	Walk	F	Left	74	35	10	134	50	+7	2	+1
6	Stroke	Walk	M	Left	38	66	11	90	63	+1	3	+1
7	Stroke	Wheelchair	M	Right	64	28	9	164	56	+3	0	0
8	Brain tumor	Walk	M	Left	34	28	11	84	64	0	4	0
9	Stroke	Walk	F	Left	72	30	7	116	48	+5	2	+2
10	Brain tumor	Wheelchair	F	Left	68	30	9	274	48	+9	0	0
11	Brain tumor	Walk	F	Left	55	28	9	152	57	0	4	0
Mean					56.3	39.5	9.3	176.6	55.5	3.6	2.2	0.5
*SD*					13.1	23	1.5	85.3	6.3	4.0	1.7	0.7

*Locomotion describes the patients' mobility (walker or wheelchair user), Rehab is the rehabilitation duration, Rec. is the number of study recording days, DAS are the days after stroke or brain tumor extraction (duration between stroke event or brain tumor extraction and rehabilitation entry). EBI scores at rehabilitation entry are denoted as: EBI^⋆^ compl for complete assessment; EBI^⋆^ walk for subcategory walking. EBI score differences between the first and last assessment are denoted as: Δ_compl_ for complete assessment; Δ_walk_ for subcategory walking*.

The EBI is a clinical assessment, primarily used to estimate the level of independence in accomplishing daily activities and divided in 16 categories including mobility (walking and stairs), transfers (e.g., bed to chair and back), feeding, dressing, grooming, and similar (Prosiegel et al., 1996). Each category was scored, using a scale from zero (patients' need full support) to four (patients' are independent of any support). The EBI was estimated twice, first at the begin of the rehabilitation as outpatient, second shortly before the patient's final discharge. In this observation study no therapy interventions of pathological gait changes and subsequent analysis were intended.

#### Study design

Each patient at the day-care centre received a personalized therapy schedule according to their expected needs, considering the level of independence and health state. The day-care centre specialized on therapy programmes, promoting the re-integration of patients into independent free-living. Patients followed their daily routines, which was partly determined by their therapy schedule, but included free time too. Patients were accompanied and observed by the study examiner for 2–3 times a week. The examiner followed the patients for up to 8 h per recording day and annotated activities online with the Android open-source smartphone framework CRNTC+ (Spina et al., 2013). Activity annotations were approved by two examiners after data post-processing. Scheduled therapies were not interrupted or skipped and all annotations were in agreement with the patient and clinicians. The study time spread over 1–3 months (39.5 day on average) including 9.3 recording days on average per patient and a total of 102 recording days. For the study, we defined an annotation catalog with a total of 51 activities, including activity primitives, *walking, walking up/downstairs, arm and leg flexion/extension, arm rotation, writing, using phone, drinking*, and similar, to describe the patients activities. For the present investigation, only walking annotations were used in further analysis. Typical, activity routines such as *eating/leisure, cognitive training, medical fitness, kitchen work, motor training, and resting* were defined as reference for potential subsequent behavior description. In the present analysis, we focus on walking and subsequent mobility behavior, and use the activity annotations for validation only. Patient therapies and incidents were documented daily in case reports according clinical guidelines, including strolls, sports, resting phases or when patients felt unwell.

#### Sensors and data recording

**Figure 1:**
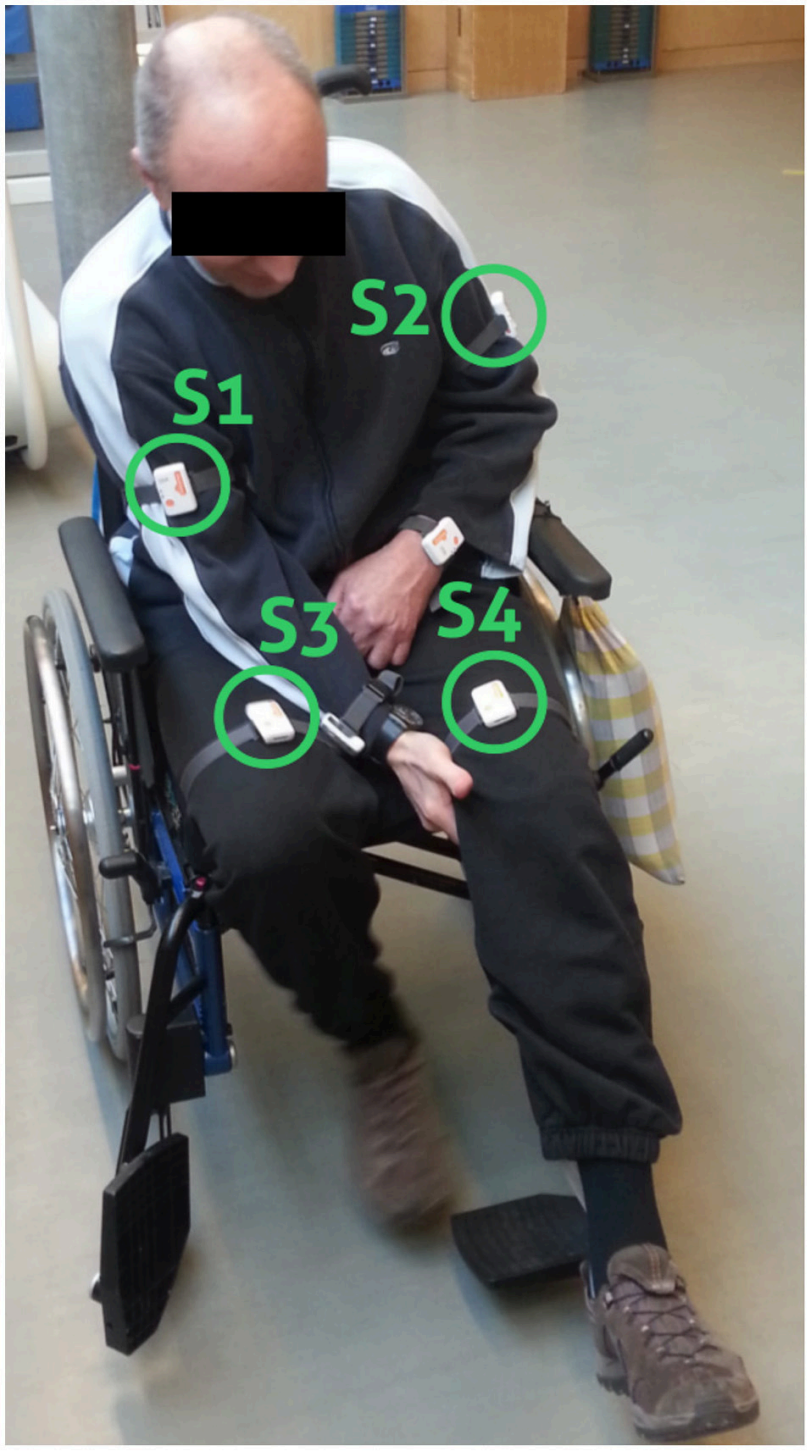
Sensor placement. Wheelchair patient with sensor positions highlighted (S1, S2, S3, and S4). Data from the wrist-worn sensors were not considered in the present analysis.

Shimmer3 IMU sensors, including a 3-axis accelerometer, a 3-axis gyroscope, and 3-axis magnetometer^1^ were attached to the patients when they arrived at the clinic in the morning. We defined sensor positions and orientation to guarantee a fixed reference system, aligning to patients' movements in x-, y-, and z-axis. Sensor position and orientation were regularly checked during recordings to avoid variation in orientation estimates or measurement offsets. Shimmer IMUs are small in size (L × W × H = 51 × 34 × 14 mm^3^), thus suitable for day-long recordings. Shimmer IMUs were configured to log sensor measurements with a sampling frequency of 50 Hz to the internal SD-card. The accelerometers range was set to ± 4 g. Sensors were attached to both wrists, upper arms, and thighs using Velcro straps. For the analysis of the walking behavior, including the upper body sway, and the subsequent bilateral trend analysis only thigh and upper arm sensors were considered necessary as illustrated in Figure 1. Our pre-investigations showed that accelerometer data provide sufficient information for unsupervised EBI score estimation (Derungs et al., 2015) and activity routine discovery in hemiparetic patients (Seiter et al., 2015). Moreover, the recent systematic review of wearable sensors for walking parameter estimation by Taborri et al. (2016), showed that accelerometer data provide sufficient information for walking segment extraction and stride segmentation. Using orientation estimates, which require all IMU sensor modalities, did not show advantages. Moreover, accelerometers are more power-efficient compared with gyroscopes, thus ideal for longitudinal studies and long-term monitoring applications. Sensors were only temporary removed during the recording day for special therapies (e.g., lymph drainage massages or water therapies) and finally detached at the end of the recording day.

### Bilateral trend analysis

**Figure 2:**
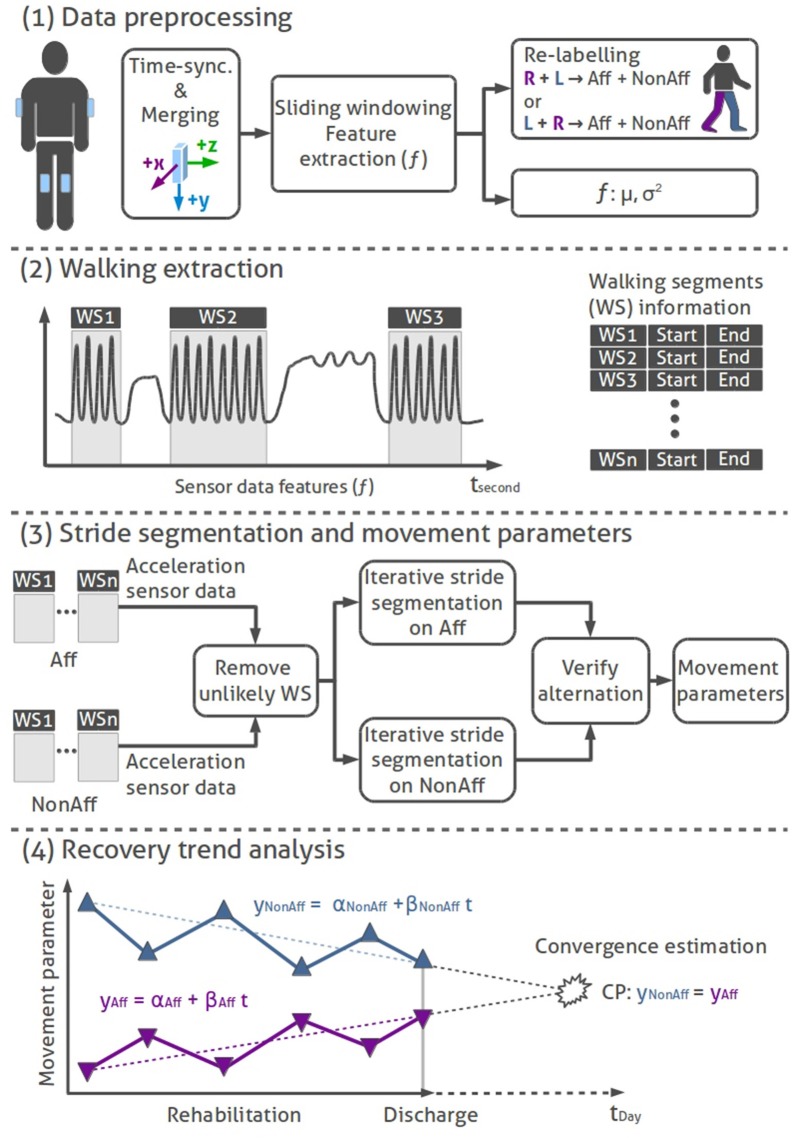
Bilateral trend analysis stages: (1) Data preprocessing: time-synchronization, merging, feature extraction, and re-labeling of IMU sensor data. (2) Walking extraction: Localizing of walking segments using logic rules. (3) Stride segmentation and movement parameters: Removing unlikely WS, stride segmentation (hill climb algorithm) on sensor data of affected and non-affected sides, alternating stride sequence verification, and movement parameter calculation. (4) Illustration of a sequential recovery trend analysis: conceptual representation of movement parameters across the rehabilitation duration. Linear trend lines on the affected and non-affected side are used to derive convergence points per movement parameter.

For our bilateral trend analysis approach we implemented a state-of-the-art data processing tool-chain to derive and evaluate movement parameters. Our four-stage approach for the bilateral trend analysis consists of (1) data preprocessing, (2) walking segment extraction, (3) stride segmentation including movement parameter extraction, and (4) regression-based recovery trend analysis. All data processing and analysis were done using MATLAB^2^. Figure 2 illustrates the walking analysis. Subsequently, each processing stage is described.

1. **Data preprocessing:** We time-synchronized and merged the data of all body-worn IMUs based on their time stamps. A non-overlapping sliding windowing of 1 s (50 samples), was applied to both thigh-worn sensors raw inertial data, to extract acceleration features *f*, including mean (μ_*k*_) and variance ($\sigma_{k}^{2}$), where *k* represents the sensors x-, y-, and z-axis. Sensor data of the corresponding body side were re-labeled with *Aff* (affected side, impaired by the stroke or brain tumor extraction) and *NonAff* (non-affected, healthy side).
2. **Walking extraction:** The walking extraction method was used to localize and select sensor data segments in walkers and wheelchair users, which likely contain walking. To extract potential walking segments (WS), we applied a logic equation to previously derived acceleration features *f*, according the indicator function 𝟙_WS_ in Equation 1.
  (1) $$\begin{array}{r} \begin{array}{c} {𝟙}_{\text{WS}}=\mu_{\text{y},\text{NonAff}}>{\text{θ}}_{1}\wedge \mu_{\text{z},\text{NonAff}}<{\text{θ}}_{2}\wedge \sigma_{\text{y},\text{NonAff}}^{2}>{\text{θ}}_{3} \end{array} \end{array}$$

NonAff denotes the non-affected side, indices y an z describe the vertical and anteroposterior axis, respectively. Thresholds were derived experimentally according to the method described in Derungs et al. (2015) by evaluating data of all participants. Thresholds θ_1_ and θ_2_ were 8 $\frac{m}{s^{2}}$ and 4 $\frac{m}{s^{2}}$, respectively. Threshold θ_3_ were 0.25 ${\left(\frac{m}{s^{2}}\right)}^{2}$ for wheelchair users and 0.7 ${\left(\frac{m}{s^{2}}\right)}^{2}$ for walkers. Due to the coarse parameter settings, we do not expect performance changes for patients, who have not been analyzed in the present dataset. In addition, viability of thigh-worn sensors for step detection has been demonstrated by Godfrey et al. (2016). Due to the higher acceleration, we used sensor data from the non-affected side to derive walking segments within all day-long recordings.

**(3) Stride segmentation and movement parameters:** Selected walking segments were further processed to derive individual strides and movement parameters. We derived the following movement parameters: stride count, normalized stride count (sum of all derived strides during the day divided by the recording duration), and the mean and standard deviation (*SD*) of stride duration, cadence, and sway. To analyse natural walking, we omitted walking segments derived during physiotherapy sessions.

We used an autocorrelation filter to remove unlikely walking segments, a second filter excluded erratic walking segments containing less than five consecutive strides. Next, walking segments were filtered using a median filter. Peak detection was applied on both thighs' acceleration data derivative using an iterative *Hill-climber* approach to find the highest peaks that segments individual strides (Aminian et al., 1999). The resulting strides of both thighs were verified with an iterative algorithm. Similar to the previous walking extraction, the algorithm used the higher acceleration signal amplitude of the non-affected thigh to locate the first stride. Starting with the first stride, the algorithm iterated over all strides on the non-affected side. In each iteration the algorithm ensured that one peak, maximum of the affected sides' acceleration data, was found. Additional peaks with lower amplitudes, detected on the affected side, were removed. The last stride could be found on either side, thus the resulting stride count for the affected and non-affected body sides could differ by one stride per walking segment. The stride duration was limited by upper (3 s) and lower (0.25 s) boundaries.

Subsequently, the cadence (C) for the affected and non-affected sides were calculated for every walking segment according to:

(2) $$\begin{array}{r} \text{C}=\frac{{\text{n}}_{\text{Strides}}}{{\text{t}}_{\text{WS}}} \end{array}$$

where n_Strides_ refers to the number of strides within the walking segment and t_WS_ is the duration of the walking segment. We defined the cadence as strides per minute according to Whittle (2007). Cadence, which is inversely proportional to stride duration, was calculated as additional movement parameter for comparison to related work. Sway (S) was extracted from the upper arm sensors' acceleration data according to the isolated strides. Sway was derived for each body side individually as lateral upper body movement perpendicular to the anteroposterior walking direction as sum of the positive and negative acceleration within each stride according to:

(3) $$\begin{array}{r} \text{S}=\frac{\sum_{\text{i}=1}^{\text{n}}\left({\text{x}}_{\text{i}}>0+{\text{x}}_{\text{i}}<0\right)}{\text{n}} \end{array}$$

where x_i_ denote acceleration samples along the x-axis and n the total number of samples within a stride.

**(4) Recovery trend analysis:** Movement parameters were analyzed as sequential observations over the rehabilitation duration, to investigate differences in daily walking behavior. Recovery trends were evaluated by linear regression models, applied to movement parameters (mean and *SD*) of affected and non-affected sides. The *SD* was used to illustrate the variability of each movement parameter across multiple recordings during the longitudinal rehabilitation. Regression slopes between body sides determined if recovery trends converge (difference between body sides decrease), diverge (difference between body sides increase) or remain parallel during the rehabilitation. The recovery trend was considered parallel, if Pearson correlation between body sides was positive and the significance level *p* < 0.05. Convergence (CP) was estimated while extrapolating and comparing recovery trends using the time variable t according to Equation 4. The regression models were: y_NonAff_ = α_NonAff_+β_NonAff_t (non-affected side) and y_Aff_ = α_Aff_+β_Aff_t (affected side) where all parameters α and β denote the regression models' offsets and slopes; t_max_ = 3,650 days (10 years).

(4) $$\begin{array}{r} \begin{array}{c} \text{CP}:{\text{α}}_{\text{NonAff}}+{\text{β}}_{\text{NonAff}}\text{t}={\text{α}}_{\text{Aff}}+{\text{β}}_{\text{Aff}}\text{t}\\ \text{ for t}=1,\ldots ,{\text{t}}_{\text{max}} \end{array} \end{array}$$

Convergence, expressed in days, quantifies movement parameter similarity. Recovery trends between affected and non-affected body sides can be considered as measure of training need. A cut-off limit of 10 years was used to indicate converging, parallel or diverging recovery trends. Continuous convergence estimates were derived using a growing window of recording days to determine movement parameter recovery trends, starting at two days. The recovery trends' goodness of fit were evaluated using *R*^2^-values. To determine statistical significance of differences between body sides and movement parameters, T-tests (*p* < 0.05) were used.

## Evaluation methods

We initially evaluated the performance of the walking extraction and the subsequent stride segmentation algorithm to derive movement parameters.

### Walking extraction

We validated the walking detection using recordings where corresponding walking annotations were available i.e., 69 out of 102 recorded days for all patients, including 54 of 63 days for walking patients, and 15 of 39 days for wheelchair dependent patients. Sensitivity and specificity were determined according to truth table quantities. Specifically, true positive (TP) denotes walking detection when annotated, true negative (TN) no walking detected when no walking annotated, false positive (FP) walking detected when not annotated, and false negative (FN) no walking detected when annotated. The walking extraction was evaluated using sensitivity and specificity. Sensitivity ($\frac{\text{TP}}{\text{TP}+\text{FN}}$) was used to evaluate that the walking detection was sensitive across all patients while specificity ($\frac{\text{TN}}{\text{TN}+\text{FP}}$) was used to rule out non-walking activities. In contrast to precision, which is referenced to the positive class, specificity is referenced to the negative class, here non-walking segments. A correct representation of the non-walking segments are of primary interest for the movement parameter analysis.

### Movement parameter validation

**Figure 3:**
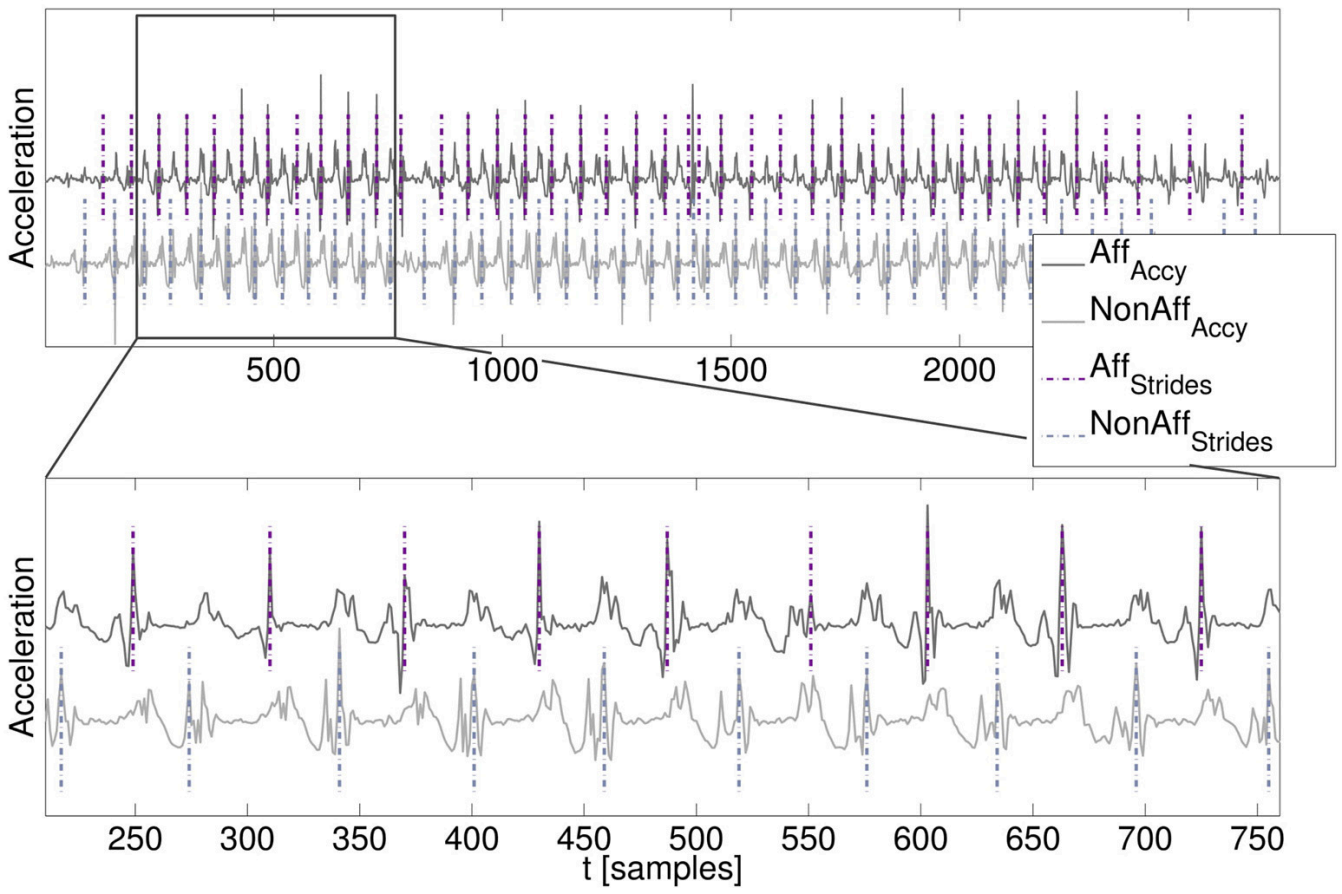
Walking segment with stride segmentation. **(Top)** Acceleration time series signals of the affected and non-affected side including signal offsets for visualization. **(Bottom)** Zoomed-in walking segment including algorithm-derived stride segmentation (dashed lines).

A manual stride annotation was performed by an expert to obtain a movement parameter reference. For each patient a walking segment was chosen from the day where patients walked most. Manual stride annotation was performed offline using MATLAB's *ginput* function. Characteristic acceleration peaks were used as annotation criteria. Resulting stride duration for individual strides were saved and subsequently the average stride duration within the walking segment was calculated. Manual stride annotation was repeated once by the same expert for the same walking segments of each patient to evaluate intra-rater variation. The automatically derived movement parameters stride count, stride duration, and cadence were subsequently compared with the movement parameter reference according to the expert's manual annotation. An example of the acceleration data and the resulting stride segmentation derived by our algorithm is illustrated in Figure 3.

### Bilateral trend analysis

Movement parameters were visualized as sequential observations across the rehabilitation duration. The movement parameter visualization provides an overview on the patients' daily variation and could be used by clinicians to interpret walking behavior. The normalized stride count, related with patients' mobility, shows the extent and variability of daily stride performances. For consistency with all movement parameters in this investigation, we present values derived from affected and non-affected body sides. Differences in stride count between affected and non-affected side were not considered as indication for recovery.

## Results

We present results of the walking extraction and movement parameter analysis including data derived from all study participants. Subsection *Bilateral trend analysis* includes information from case reports to detail walking behavior of three patients exemplarily.

### Walking extraction

Extracting walking segments from the patients' data is essential to subsequently estimate movement parameters. Therefore we validated the walking segment extraction performance. Table 2 summarizes the truth table quantities of the walking segment extraction. On average, recorded movement data provided 3.45 % (all patients), 4.42 % (walkers), and 1.75 % (wheelchair users) walking annotations for the validation. Sensitivity was on average 69.3 % (including all patients). Sensitivity for walkers was 79.9% and 50.7 % for wheelchair users. Generally, increased sensitivity was found for walkers, due to higher mobility and walking events, compared to wheelchair users. Average specificity was greater than 94 %, confirming that extracted walking segments were mostly correct. Results showed that the unsupervised walking segment extraction was sensitive to walking segments of walkers and wheelchair users even when only few walking events occurred.

**Table 2:** Validation results of the walking extraction.

**ID**	**TP (%)**	**TN (%)**	**FP (%)**	**FN (%)**	**Sens (%)**.	**Spec (%)**.	**Annot. (days)**	**Rec. (days)**
1	1.49	96.15	0.44	1.92	40.22	99.54	4	11
2	4.79	87.04	5.92	2.25	69.99	93.64	5	8
3	0.27	96.30	3.25	0.18	63.11	96.74	3	10
4	1.81	94.03	3.02	1.14	76.17	96.94	6	7
5	2.60	92.52	4.06	0.83	72.30	95.80	10	10
6	3.76	88.68	6.72	0.84	86.80	93.02	10	11
7	0.06	99.01	0.90	0.02	75.88	99.10	3	9
8	2.07	89.23	8.56	0.15	88.83	91.28	8	11
9	4.73	90.38	3.82	1.07	80.91	95.95	7	7
10	0.70	96.64	0.30	2.36	23.55	99.69	5	9
11	4.12	88.21	6.86	0.81	84.29	92.82	8	9
	Average—All patients						Sum	
	2.40	92.56	3.99	1.05	69.28	95.87	69	102
	Average—Walker (IDs: 2, 4, 5, 6, 8, 9, 11)						Sum	
	3.41	90.01	5.57	1.01	79.90	94.21	54	63
	Average—Wheelchair (IDs: 1, 3, 7, 10)						Sum	
	0.63	97.03	1.22	1.12	50.69	98.77	15	39

*Truth table quantities per patient are shown. The validation was performed for available walking annotations. Sens., sensitivity of the walking extraction; Spec., specificity of the walking extraction rejecting non-walking activities; Annot., recording days that include walking annotations; Rec., total recording days*.

### Movement parameters

Validation results and relative errors are summarized in Table 3. Comparing the automatic stride segmentation with the experts' manual stride reference, the following average relative errors were found; 2.26 % (stride duration), −2.76 % (cadence), and 3.67 % (stride count) at the affected side; 3.96 % (stride duration), −5.41 % (cadence), and −0.79 % (stride count) at the non-affected side. The underestimation of average cadence values was attributed to a biased manual walking segment extraction based on the visual interpretation of walking segment boundaries. Higher deviations were found for patient ID1 (28.10 %, stride duration) and ID8 (−20.82 %, cadence). For ID1 only few strides could be used for the segmentation, rendering the statistics unreliable. For patient ID8 the cadence was high, while the stride duration was low, suggesting that the patient walked fast but with short stride length. The repetition of the manual stride annotation by the same expert yielded average intra-rater changes below 1 %, thus we consider the manual reference reliable. Relative errors for movement parameters stride count, stride duration, and cadence, were below 6 % on average. We attributed the errors to the few falsely detected strides. Average differences between affected and non-affected sides were; 0.18 strides (stride count), 0.07 strides/min (cadence), and 0 s (stride duration) using a manual stride segmentation. Using the automatic stride segmentation, differences were 1 strides (stride count), 0.69 strides/min (cadence), and 30 ms (stride duration).

**Table 3:** Validation results of the algorithm-derived movement parameters compared with the experts' derived manual stride reference.

	**Experts' manually derived movement parameters**						**Algorithm-derived movement parameters**						**Relative error (%)**					
	**Stride duration (s)**		**Cadence** $\left(\frac{\text{strides}}{\text{min}}\right)$		**Stride count (strides)**		**Stride duration (s)**		**Cadence** $\left(\frac{\text{strides}}{\text{min}}\right)$		**Stride count (strides)**		**Stride duration (s)**		**Cadence** $\left(\frac{\text{strides}}{\text{min}}\right)$		**Stride count (strides)**	
**ID**	**Aff**	**NonAff**	**Aff**	**NonAff**	**Aff**	**NonAff**	**Aff**	**NonAff**	**Aff**	**NonAff**	**Aff**	**NonAff**	**Aff**	**NonAff**	**Aff**	**NonAff**	**Aff**	**NonAff**
1	2.09	2.10	32.37	28.78	9	8	1.72	1.51	28.40	31.80	8	9	17.70	28.10	12.26	−10.49	11.11	−12.50
2	1.17	1.18	50.96	51.46	103	104	1.14	1.13	53.26	53.41	104	105	2.56	4.24	−4.51	−3.79	−0.97	−0.96
3	1.24	1.24	46.36	46.36	53	53	1.23	1.24	45.94	46.20	50	51	0.81	0.00	0.91	0.35	5.66	3.77
4	1.06	1.06	56.76	56.76	76	76	1.07	1.06	56.42	56.82	75	76	−0.94	0.00	0.60	−0.11	1.32	0.00
5	1.34	1.34	45.21	44.73	93	92	1.38	1.36	44.23	44.46	88	89	−2.99	−1.49	2.17	0.60	5.38	3.26
6	1.30	1.27	46.19	47.37	39	40	1.28	1.23	49.34	49.76	40	41	1.54	3.15	−6.82	−5.05	−2.56	−2.50
7	1.92	1.92	32.48	32.48	11	11	1.91	1.92	34.17	35.33	10	11	0.52	0.00	−5.20	−8.77	9.09	0.00
8	1.14	1.14	50.63	51.71	47	48	1.07	1.07	61.17	61.38	48	49	6.14	6.14	−20.82	−18.70	−2.13	−2.08
9	1.26	1.27	47.60	47.60	86	86	1.29	1.29	47.75	48.05	83	84	−2.38	−1.57	−0.32	−0.95	3.49	2.33
10	2.76	2.73	21.96	21.96	10	10	2.73	2.68	22.80	23.89	9	10	1.09	1.83	−3.83	−8.79	10.00	0.00
11	1.26	1.27	47.12	47.60	98	99	1.25	1.23	49.40	49.40	98	99	0.79	3.15	−4.84	−3.78	0.00	0.00
Avg.	1.50	1.50	43.42	43.35	56.82	57.00	1.46	1.43	44.81	45.50	55.73	56.73	2.26	3.96	−2.76	−5.41	3.67	−0.79

*For each patient one walking segment from the day with the highest stride count was selected*.

**Figure 4:**
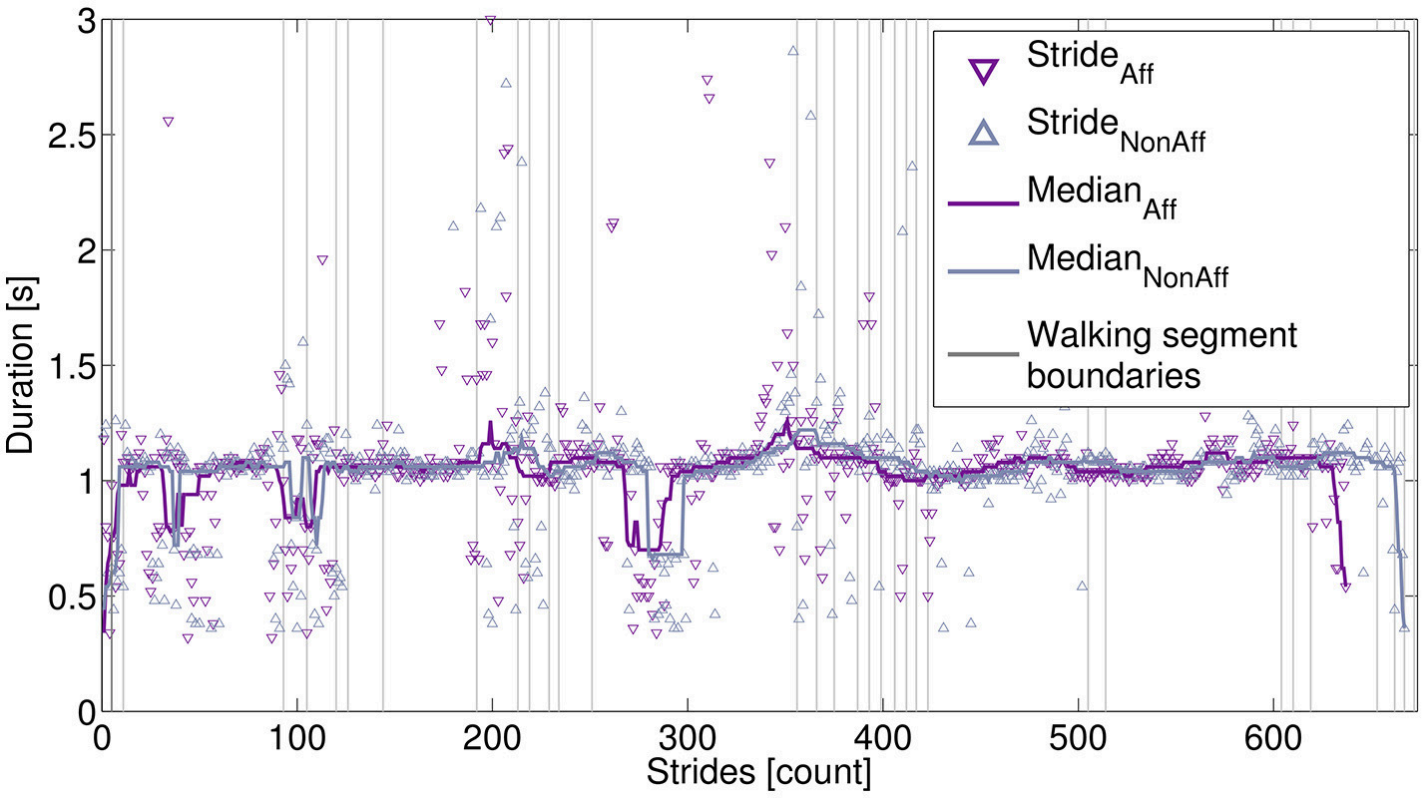
Extracted strides derived from one recording day. Strides are marked for the affected side (637 strides) and non-affected side (667 strides). The stride differences were due to the processing of walking segments, as described in the main text. Stride durations were smoothed, using a median filter. Vertical lines indicate starting and ending of individual walking segments.

Figure 4 illustrates the movement parameter stride duration derived from one recording day (patient ID6, walker). A median filter ($order=\sqrt{Strides}$) applied to the affected and non-affected strides showed that both body sides had similar stride durations, indicating a balanced walking style. The patients' walking behavior during the day, including sections, where the patient moved faster can be observed around strides 50, 100, and 280.

**Figure 5:**
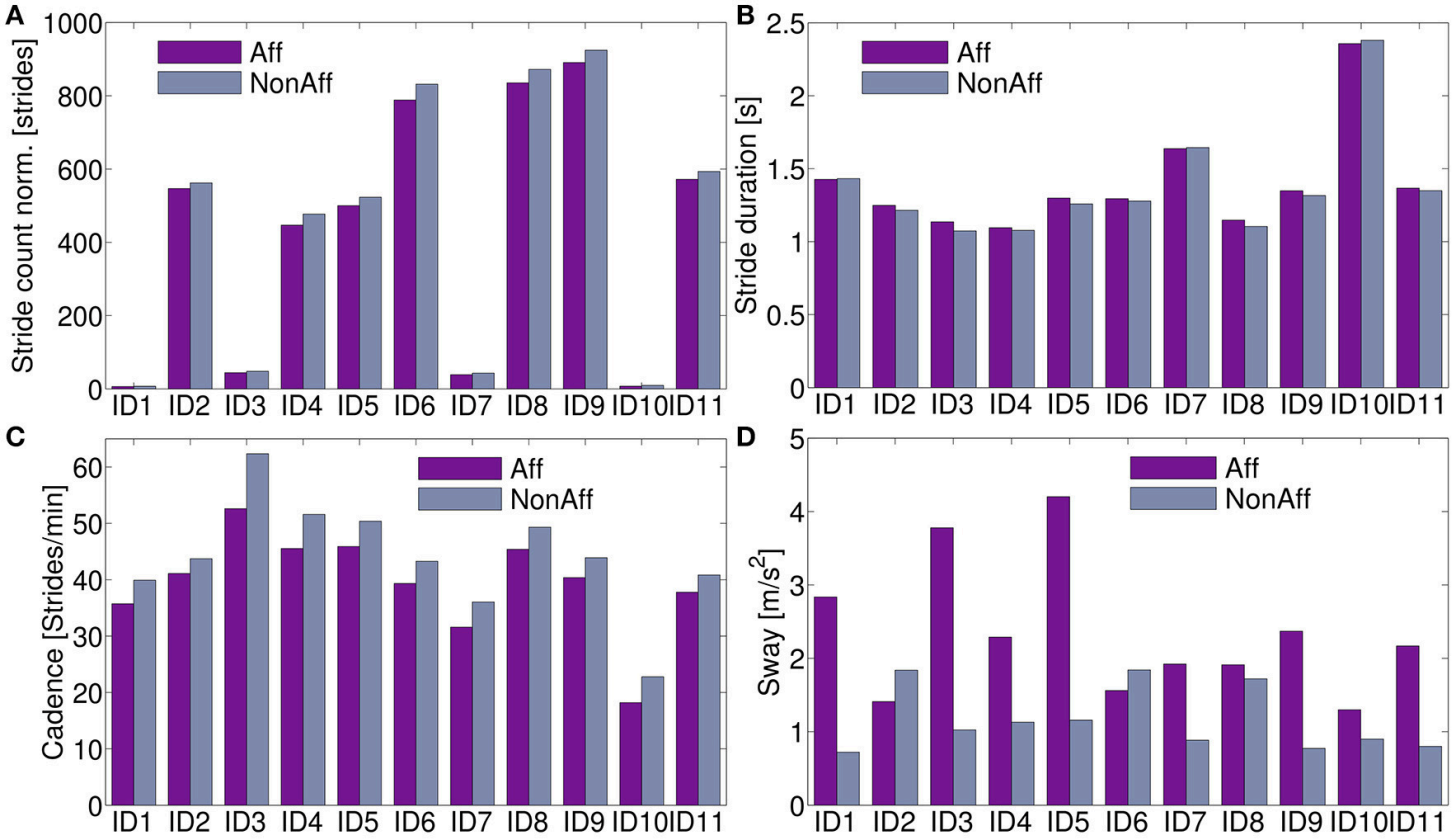
Movement parameters. **(A)** Stride count norm. over recording days; significantly more strides in walkers compared to wheelchair users were found (*p* < 7.3 × 10^−5^). **(B)** Stride duration differed on average 2.2 % between body sides. **(C)** Cadence was on average across all patients 12.2 % higher at the non-affected side. **(D)** The sway showed significant differences between body sides (*p* < 0.0011).

All movement parameters were summarized in Figure 5. For ID9 (walker), a maximum of 891 strides (affected side) and 924 strides (non-affected side) were found. Walkers, including patients ID2, ID4, ID5, ID6, ID8, ID9, and ID11, performed significantly more strides than wheelchair users (*p* < 7.3 × 10^−5^).

The stride duration and sway, showed no clear pattern to distinguish between walker and wheelchair users. Stride duration, (Figure 5B) was on average 0.6 % lower at the affected side for patients ID1, ID7, and ID10 (wheelchair users). Walkers and one wheelchair user (ID3) showed 2.8 % higher values at the affected side on average. Cadence was on average 12.2 % higher for all patients in the non-affected side (Figure 5C). For patients ID2 and ID6 the sway was significantly higher at the non-affected side (*p* = 0.042), remaining patients showed significantly higher sway in the affected side (*p* = 0.0003). Averaged movement parameters were summarized in Table 4.

**Table 4:** Summary of extracted movement parameters averaged over all patients.

		**Averaged movement parameters**				
**Body side**	**Statistic feature**	**Stride count total**	**Stride count normalized**	**Stride duration (s)**	**Cadence $\left(\frac{\text{strides}}{\text{min}}\right)$**	**Sway $\left(\frac{\text{m}}{{\text{s}}^{2}}\right)$**
Aff	Mean	3880	425	1.40	39.41	2.34
	*SD*	3372	347	0.35	9.06	0.93
	Min	66	7	1.10	18.19	1.30
	Max	9187	891	2.36	52.61	4.20
NonAff	Mean	4059	445	1.38	44.01	1.16
	SD	3521	361	0.37	10.02	0.43
	Min	79	7	1.07	22.77	0.72
	Max	9592	924	2.38	62.35	1.84

*Stride count total (sum of all strides) and normalized count (total stride count divided by recording days) showed high SD due to different walking behavior of wheelchair users and walkers*.

### Bilateral trend analysis

**Figure 6:**
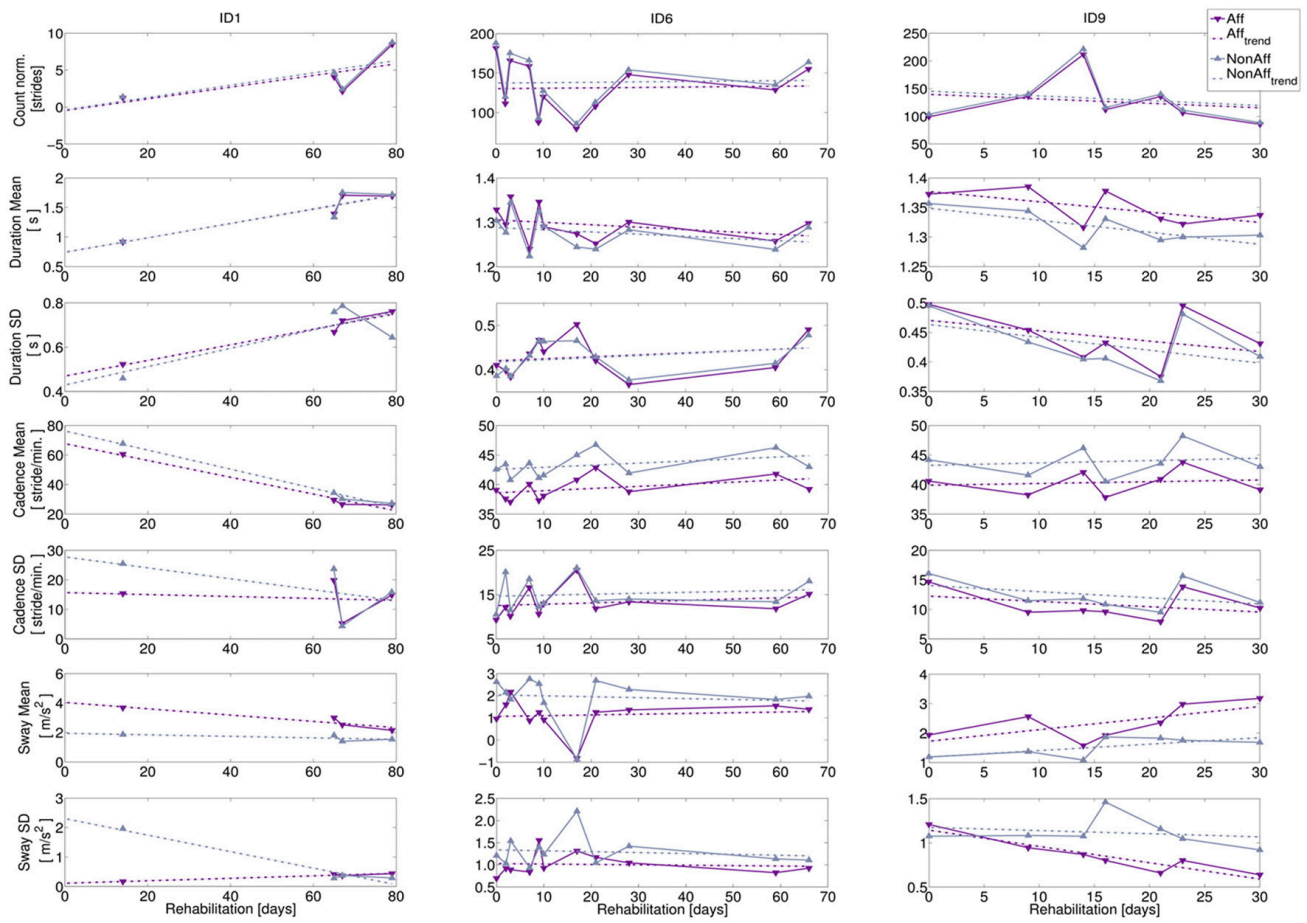
Bilateral trend analysis. Left column: wheelchair user (ID1, age = 57 years, 11 recording days), middle column: walker (ID6, age = 38 years, 11 recording days), right column: walker (ID9, age = 72 years, 7 recording days). Top to bottom: extracted movement parameters: normalized stride count, stride duration (mean and *SD*), cadence (mean and *SD*), and sway (mean and *SD*). Recording days where walking was extracted relative to study begin are indicated by markers, dashed lines indicate recovery trends.

The bilateral trend analysis across movement parameters revealed differences and variability between patients during the recording period. We subsequently present an interpretation of the recovery trends using case reports for three typical patients representing the study population: a wheelchair user (ID1) and two walkers of different age (ID6, ID9). The corresponding diagrams are shown in Figure 6, while Table 5 summarizes case report data of the patients' therapy programme. To illustrate the diversity in motion parameters across all study patients, we included remaining patient diagrams in the Appendix (Figures S1–S3 in Supplementary Material).

**Table 5:** Case reports for the three selected patients, including personal therapy schedules.

**ID**	**Day**	**Dur (h)**	Therapy schedule, (^*^ see Notes)	**Notes**
1	1	5.6	ST, ET, CT, S, R, ST, PT, IT,	
	2	7.4	ST, ET, ET, PT, S, CT, ST	
	3	6.0	ET, PT, ST, R, V, CT, IT	
	4	7.5	S, ET^*^, S, CT, R, PT^*^, S, SpT, IT	Walking attempts
	5	7.7	V, ET, S, ET, PT, S ST, S	
	6	7.8	R, PT^*^, S, ET^*^, SpT, R, SpT^*^, S	Walking attempts
	7	7.8	SpT, ET, SpT, S, R, ST, SpT, PT, R	
	8	6.9	R, ET, PT, SpT, IT	
	9	4.9	ST, DT, ST, S	Walking
	10	6.6	ST, PT, ET, ST IT	Walking
	11	3.3	ET, ST, DT	Walking
6	1	5.3	ET, DT, PT, V, ST, PT, IT	
	2	6.6	ET, DT, ET, PT, ST, IT	
	3	6.4	ST, ST, PT^*^, IT,	Walking-tests
	4	5.8	PT, ET, DT, V, ST, IT	
	5	6.9	ET, PT, DT, ST, IT	
	6	6.5	PT, ST, ST	Exhaustion
	7	6.8	PT, ST	Rests, headache
	8	7.7	PT, S, ET, PT, S, ET^*^ ST, V, IT	Walking
	9	5.7	PT, S, ET^*^	Walking, stroll
	10	5.6	PT, ST, IT, S	
	11	4.2	V, PT, IT, ET, ST	
9	1	4.5	V, PT, DT, ST	
	2	7.5	PT, ST, ET, DT, BT, V, IT	Walking
	3	6.4	ET^*^, PT, DT^*^, R, SpT, IT	Walking
	4	7.8	CT, PT, DT, BT, R, ST, V	Head bumped
	5	7.3	CT, V, PT^*^, SpT, IT	Walking exercises
	6	8.0	DT, PT, DT, BT, V, IT, ST	
	7	8.0	CT, PT, DT, BT, ET, SpT, IT	

*ET, ergotherapy; PT, physiotherapy; DT, training of ADL, e.g., lay the table; ST, self training, e.g., drawing, CT, cognitive training; IT, intense training in the gym; S, socializing; R, resting; SpT, special therapy, e.g., lymph drainage; V, visit, e.g., physician; BT, balance training*.

1. **ID1:** The patient was wheelchair-dependent, however progressed during the 79-day rehabilitation period toward an independent walker. Progress indicators can be found during recording days 9, 10, and 11 (e.g., the normalized stride count increased toward the rehabilitation discharge). The sway mean revealed a significant difference between body sides (*p* = 0.015), while the other movement parameters were balanced.
2. **ID6:** The variation in normalized stride count ranged from minimal 79 strides to a maximum of 188. Stride duration mean and SD showed no statistical significant differences during the rehabilitation, suggesting balanced walking. The cadence mean, was significantly higher in the non-affected side (*p* = 0.0001), but differences between affected and non-affected sides were stable. Cadence SD showed no significant differences. Sway mean at day 7 appeared to be an outlier.
3. **ID9:** Stride extraction varied between 86 and 221 strides per day. However, cadence mean was significantly higher at the non-affected side (*p* = 0.017), while cadence SD showed no significant difference. Moreover, movement parameter trends indicate that decreasing stride duration led to increasing cadence. On average, sway mean was 53 % higher at the affected side (significant, *p* = 0.008), although the sway SD at the affected side decreased significantly over time (*p* = 0.016), suggesting that upper body deficits affected walking pattern.

We derived *R*^2^-values of recovery trends showing fits up to 0.9, e.g., for stride duration mean (ID1) and ranging between 0.4 and 0.9 for sway (ID9). Remaining movement parameters showed *R*^2^-values lower than 0.4, caused by data variance. Patients' case reports, summarized in Table 5, indicated that daily recording durations, therapy schedules, therapy type, and transfers between therapy locations influenced daily walking durations and resulting stride count. Individual behavior and medical incidents influenced walking behavior too, as shown in the case reports. For example, ID6 (recording day 7) had two therapy sessions and suffered from a headache, thus forced resting phases, resulting in reduced walking.

**Figure 7:**
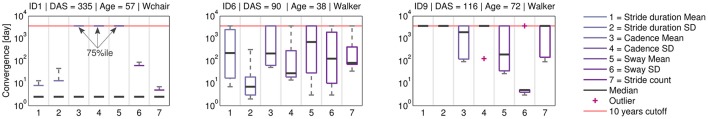
Continuous convergence estimates. Across all patients, variance was observed in the movement parameters. ID1 showed low median CP, suggesting movement parameter convergence between body sides toward discharge. For ID6 and ID9 convergence trends differ by movement parameter. The CP analysis can help to identify patient-specific therapy needs.

Figure 7 shows the continuous convergence estimation as box plots for each movement parameter. Convergence was not guaranteed and some CP's were limited by the 10 years cut-off. For example, ID1 showed increased mobility toward the end of the rehabilitation, thus convergence was reached. However, results revealed inter-patient variability within and across movement parameters.

## Discussion

### Walking extraction

The approach presented in this work investigated walking to derive movement parameters and compared body sides. Although, patients included in our study varied in age, gender, type of locomotion, and cause of impairment, which all affected walking patterns, walking extraction performance resulted in an average sensitivity of ~ 70% across all patients. The detection was sufficiently sensitive to extract walking segment from each patient even if only few walking events occurred. For the movement parameter estimation from walking, our walking segment extraction does not require perfect sensitivity. Instead, specificity must be maximized to accurately estimate movement parameters. The average specificity in our analysis was above 94%, which demonstrates that non-walking activities were mostly rejected. Further, our rule-based algorithm does not require any data-based training and could be used for remote monitoring, were data annotation is infeasible. In our investigation, sensors were placed at anatomical landmarks, i.e., above the knee, and did not require adjustment to pre-defined angles. Due to the diversity in our study population, we are confident that the coarse threshold setting used in walking segment extraction remains viable for other, yet unseen, patients with hemiparesis and could tolerate natural positioning offset and orientation variability. However, further investigations are needed to quantify the orientation variability effect and potentially develop compensation methods. Our approach promotes rapid sensor attachment and could facilitate self-attachment of sensors by patients in remote home monitoring settings without guidance, e.g., by integrating sensors in clothing. With the natural readjustment of clothing, orientation variability may be reduced too.

### Movement parameters

Wide inter- and intra-patient variability and inconsistent walking styles, including asymmetries and irregularities render stride segmentation using peak detection challenging (Parisi et al., 2016). For consistency across all movement parameters, we calculated and illustrated normalized stride counts for both body sides too. Our algorithm ensured that stride count differences between affected and non-affected body sides did not exceed one stride per individual extracted walking segment. Consequently, summation of walking segments per recording day could result in stride count differences grater one. Stride count differences were not considered as recovery trend indication. The stride count parameter was intended to illustrate extent and variation in daily movement and mobility behavior.

Our choice of movement parameters and analysis of differences between body sides is motivated by patients' compensation strategies. Patients with a hemiparesis, e.g., after stroke, often develop compensation strategies including shoulder and trunk rotations to cope with functional limitations and moreover show disturbed balance, which is influencing movement of affected and non-affected body sides (Di Fabio et al., 1986; Bourbonnais and Noven, 1989). Related work involving measurements in controlled settings further showed that patients with a hemiparesis may develop abnormal walking patterns due to muscle weakness (de Quervain et al., 1996; Chen et al., 2005). Abnormal walking patterns and asymmetries could influence all phases of the gait cycle including stride duration (sum of swing- and stand-duration), stride velocity, cadence, and similar (Titianova et al., 2003; Moe-Nilssen and Helbostad, 2004; Patterson et al., 2010). Although stride duration and cadence are confirmed as relevant movement parameters for recovery indication in the literature, results from our free-living study showed no clear recovery trends in movement parameters. Stride parameter related to impairments of patients after stroke, e.g., scuffing, fatigue, or imbalance could be investigated to further understand the recovery process. However, in the present observation study such extended parameter analysis were not intended.

Advanced stride segmentation algorithms to derive movement parameter exist, however, validation were restricted to lab-controlled settings. How such algorithms perform in free-living remains unclear, hence our approach utilizes established methods for subsequent bilateral movement analysis. Although, the extent of our validation was limited, and false strides could be detected, we found that our averaged movement parameters stride duration (1.40 ± 0.35 s affected side, 1.38 ± 0.37 s non-affected side) and cadence (39.41 ± 9.06 strides/min affected side, 44.01 ± 10.02 strides/min non-affected side), were similar to published values. In a lab recording study with six hemiparetic patients after stroke, (Chen et al., 2005) measured stride durations of 1.47 ± 0.21 s and cadences of 83.4 ± 12.8 steps/min. Cadence was expressed in steps per minute, thus the value is doubled, compared to strides per minute. Moreover, Chen et al. showed that movement parameters (stride duration and cadence) in patients after stroke were similar to healthy controls. Fraccaro et al. (2014) found averaged stride duration of (1.1 – 1.18 s) and cadence of (99.62 –129.07 steps/min) while analyzing healthy older adults using Shimmer sensors. Further, the validation of automatically derived movement parameters with the experts' manual stride reference renders the influence of falsely detected strides on the subsequent recovery trend analysis negligible.

Sway was determined from bilateral upper arm sensors as variation of the trunk position perpendicular to the walking direction. We expected that the upper arm position is advantageous regarding sensor wearing comfort and provides larger sway amplitude compared to a waist-worn sensor that is closer to the centre of mass. Wearing comfort is crucial for day-long recordings. In particular, sensors at the lower trunk might affect the patients' activities, e.g., when using toilets, sitting, or resting. Orientation variability could result in reduced acceleration amplitudes and thus modify the sway estimate. Similar to the thigh sensor position, visual inspection during the recordings did not reveal a large orientation variation of the upper arm sensors and no readjustment was made during recordings. Nevertheless, further evaluations are needed, e.g., using an optical reference system, to investigate sensor alignment and the effect on sway amplitude. Based on garment fitting simulations, Harms et al. (2012) showed that the angular variability of clothing-integrated sensors and closely fitting garments can remain below 15°, thus would constrain sway amplitude variation below ± 5%. Different strategies were described in the literature to reduce the influence of a varying sensor orientation. For example, Kunze and Lukowicz (2008) proposed a combination of acceleration and gyroscope data to compensate for sensor placement variances. However, for long-term recordings, gyroscopes are inappropriate due to the increased power-consumption compared to accelerometers.

### Bilateral trend analysis

The proposed recovery trend analysis of the patients' movement parameters over several months including affected and non-affected body sides in realistic day-care settings were not addressed in the literature so far. Our study, including 11 patients and 102 day-long recordings revealed differences between patients and variability in movement parameters. The wheelchair users in this study reflected the patient continuum from initial wheelchair dependency toward independent walking. In particular, patients ID1, ID3, ID7, and ID10, were transforming from wheelchair users to walkers during the study duration. Objective sensor measurements could provide clinicians valuable information about mobility behavior and recovery trends in unsupervised remote monitoring, even when patients are at the boundary to walking independence and yet rarely walk. Although van Meulen et al. (2016) proposed evaluation metrics for daily life movements, only 201 min of movement data derived from two patients were analyzed. For the approach presented in this current study, recordings across several days were used to extract walking segments and analyse movement parameters.

Typically, analysis of walking-related movement parameters used feet, ankle, or trunk mounted sensors (Aminian et al., 1999; Moe-Nilssen and Helbostad, 2004; Zijlstra, 2004). The present study aimed at an unobtrusive measurement during daily activities by choosing sensor positions at thighs and upper arms, hence suited for exercise monitoring in remote settings too. Advantages of thigh-worn sensors, e.g., for posture analysis were further discussed by Godfrey et al. (2016). Nevertheless practical sensor mounting and integration must be considered. We believe that the selected positions could be realized, e.g., using unobtrusive shirt- and trouser-integrated sensors, similar to the approach described by Tognetti et al. (2005). Hence, patients with a hemiparesis may not need to handle sensors separately from their clothes.

Analyzing rehabilitation patient progress is an open challenge. While clinical assessments require patients to perform specific exercises, our approach was to interpret daily free-living activity, specifically walking.

For post acute stroke patients, the relation of affected and non-affected body sides was first investigated by Gubbi et al. (2013). When comparing body sides, individual anthropometric characteristics are considered and an absolute reference to an “ideal” movement pattern is avoided. The present investigation showed for the first time results in an unconstrained setting. By considering walking segments, a natural and repeatable assessment condition for movement parameters is created, applicable outside of clinical settings and assessments. Patients' daily stride counts, which is related to walking behavior and mobility, were compared against case reports for three typical patients, as representatives of the study population, including a wheelchair user and two walkers of different age. Based on clinical case reports, we found that the sensor-reported stride count was related with type and amount of scheduled therapies. We believe that clinicians could benefit from quantified movement analysis using wearable sensors to devise therapy. The presented analysis could facilitate remote monitoring applications, where patients follow their activities in home environments.

The patient recovery progress was expressed as convergence of individual movement parameters between body sides, and quantified as CP estimate. The impairment of the affected side typically results in compensations performed by the non-affected side. We therefore expected to see changes in movement parameters of both body sides. Due to the gradual stroke recovery process over weeks, months, and even years, we used linear regressions to describe convergence trends. We consider the CP as useful metric for remote patient monitoring, where CPs could indicate patient-specific therapy needs. In the present study, therapy strategies were still chosen independent of CP estimations. In the future, CPs estimated on each day could contribute to specific, individualized therapy choices. Personal therapy plans could be created in the clinic as well as at home, e.g., by selecting exercises that address movement parameters with relatively large CP estimates and continue to adjust the personalized training programme as the patient's CP estimates change. For example, walking could be promoted by treadmill training or outdoor strolls, improving the movement parameters stride duration and cadence. Sway could be improved via balance gaming exercises (Morone et al., 2014).

For some patients, e.g., ID1, convergence was found during the rehabilitation period. For other patients and movement parameters, both, recovery trends and CPs varied. Using case reports, some influence of the daily therapy programme and special events could be identified. Another source of variation are mood and social stimuli, which were not monitored in the current study. In contrast, the influence of falsely detected strides for the movement parameter calculation and subsequent convergence point estimation was considered negligible. By narrowing the activity, e.g., walking at a given speed (Altini et al., 2016), or analyzing selected exercises, a focused assessment condition could be realized and variability in movement parameters may be reduced. Since we extracting walking segments in free-living behavior data, we did not require patients to perform specific test exercises. On the other hand, constraining the analysis to a given walking speed would have led to narrow applicability of our approach as the walking ability of patients with a hemiparesis varies widely. Our sequential recovery trend analysis started with only two observations where outliers may affect the convergence point estimate more than after additional observations from further monitoring days were included. Based on the long-term therapy progress or changes in the training plan, it is conceivable to adjust the observation window, e.g., to include only the most recent observations.

## Conclusion

We analyzed movement parameters of patients with a hemiparesis during free walking over weeks and months and compared affected and non-affected body sides to investigate the long-term recovery process in an outpatient setting. In particular, the mobility behavior analysis and investigation of potential recovery trends were analyzed using algorithms which do not depend on manually annotated data. We demonstrated that our analysis can be used in rehabilitation settings, where patients follow their personal daily routines. Hence, our method could render remote monitoring in free-living feasible. Movement parameter ranges for stride duration and cadence confirmed previous lab study results demonstrating the usability of thigh worn sensors. However, no consistent trend across movement parameters and patients was found due to parameter variability. Case reports confirmed that variability in movement parameters was caused by patient-individual habits, amount and type of scheduled therapies, and patients' daily health conditions.

Comparing recovery trends between affected and non-affected body sides regarding the movement parameters could be done using the convergence point estimation. Although, convergence was not guaranteed in the present study, our analysis showed that convergence could be achieved. Hence, we believe that convergence points provide an intuitive view on individual patient deficits that could be assessed remotely during everyday life and help to personalize training programmes. Furthermore, we expect that our analysis can be extended to follow-up studies including additional movement parameters and generalizes to recovery trend analysis in larger hemiparetic patient populations.

## Ethics statement

Following detailed verbal and written information about the character of the study, all participants signed an informed consent according the declaration of Helsinki before the recording began. Participants further approved the publication of patient details and study results according the signed informed consent. The protocol of the study was approved by the Swiss cantonal Ethics committee of the canton Aargau, Switzerland (Application number: 2013/009).

## Author contributions

AD: data recordings, algorithm development and analysis; CS-A: supported the study design, ethics application, and approval process, helped with patient recruitment and inclusion; OA: helped in all facets of the study and manuscript preparation. All authors read and approved the final manuscript.

### Conflict of interest statement

The authors declare that the research was conducted in the absence of any commercial or financial relationships that could be construed as a potential conflict of interest.

## Footnotes

^1^Shimmer, DCU Alpha, Old Finglas Road, Glasnevin Dublin 11, Ireland.

^2^MATLAB, Release 2013b, The MathWorks, Inc., Natick, Massachusetts, United States.

## Supplementary material

The Supplementary Material for this article, including the Appendix, can be found online at: https://www.frontiersin.org/articles/10.3389/fbioe.2018.00057/full#supplementary-material
