# Supplementary material for: Longitudinal Walking Analysis in Hemiparetic Patients Using Wearable Motion Sensors: Is There Convergence Between Body Sides?
Source: Front Bioeng Biotechnol. 2018 May 31;6:57. doi: 10.3389/fbioe.2018.00057 (PMC5990601; doi:10.3389/fbioe.2018.00057)
Supplement: Supplementary file 1 [file Image_1.pdf]

Supplementary Material: Longitudinal Walking Analysis in Hemiparetic Patients using Wearable Motion Sensors: Is there convergence between body sides?

1 APPENDIX

The bilateral trend analysis of the patients ID2-4, ID5, ID7, ID8, ID10, and ID11 are summarised in Figures S1, S2 and S3.

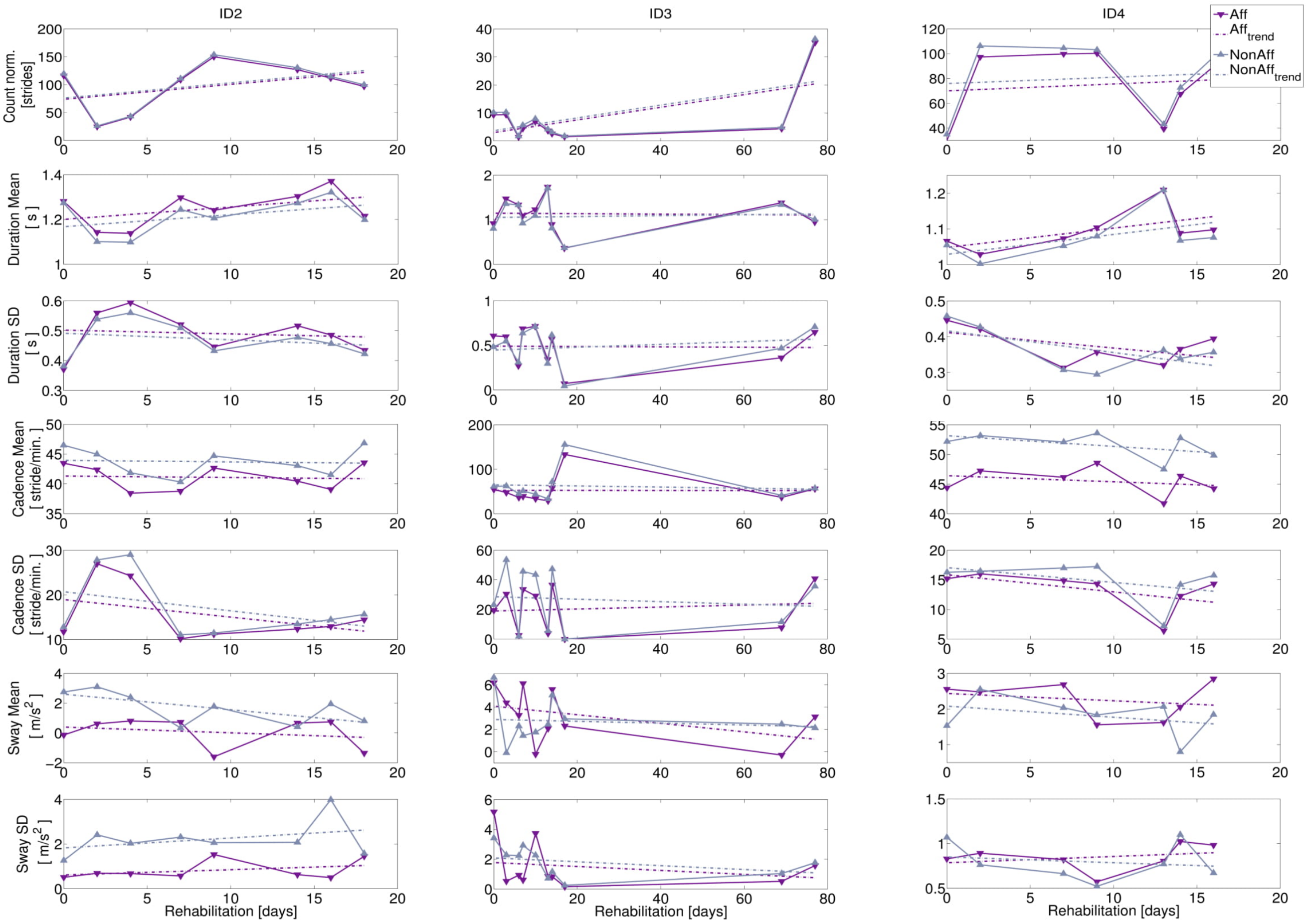

Figure S1. Bilateral trend analysis. Left column: walker (ID2, age = 47 years, 8 recording days), middle column: wheelchair user (ID3, age = 53 years, 10 recording days), right column: walker (ID4, age = 52 years, 7 recording days). Top to bottom: extracted movement parameters: normalised stride count, stride duration (mean and SD), cadence (mean and SD), and sway (mean and SD). Recording days where walking was extracted relative to study begin are indicated by markers, dashed lines indicate recovery trends.

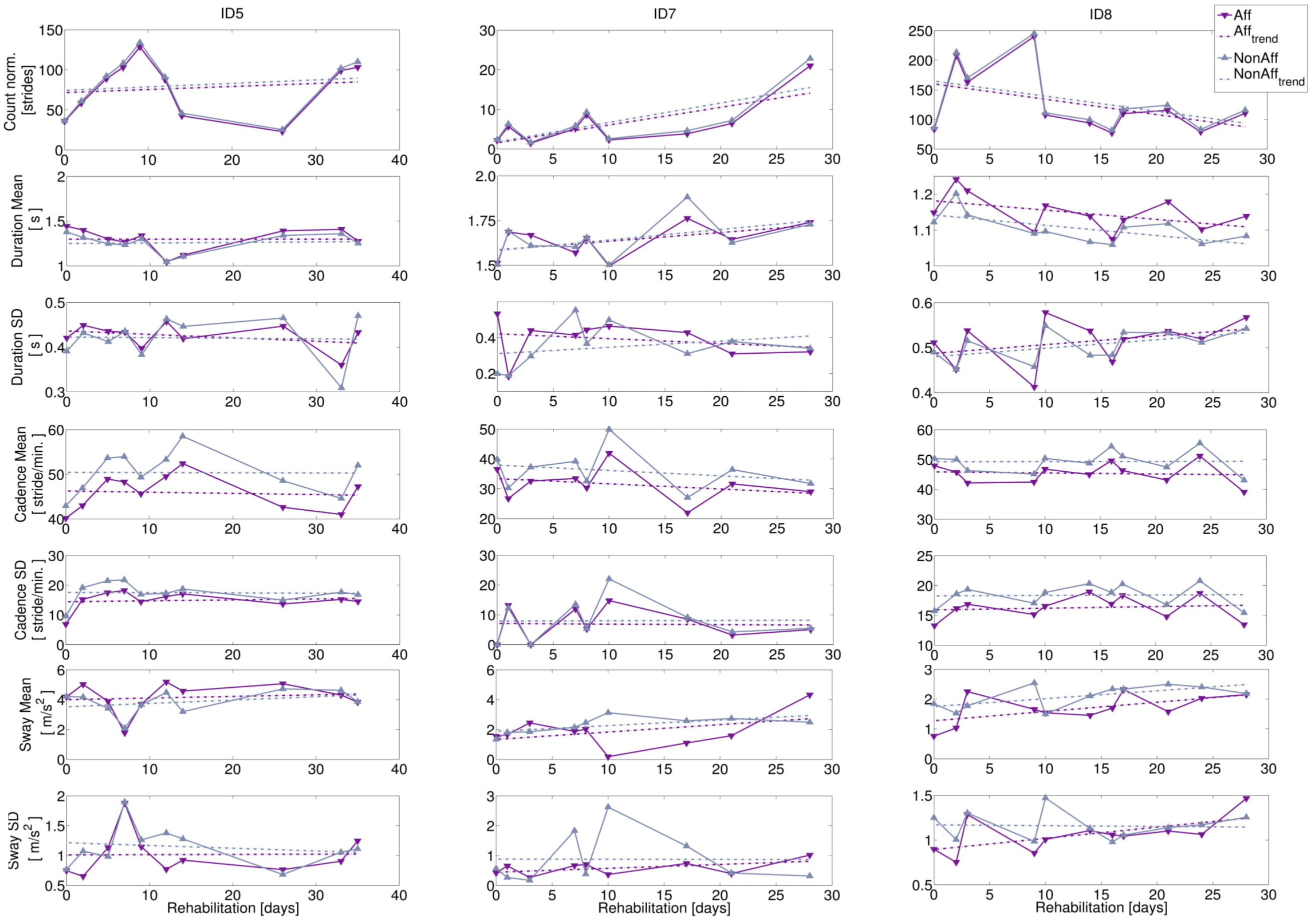

Figure S2. Bilateral trend analysis. Left column: walker (ID5, age = 74 years, 10 recording days), middle column: wheelchair user (ID7, age = 64 years, 9 recording days), right column: walker (ID8, age = 34 years, 11 recording days). Top to bottom: extracted movement parameters: normalised stride count, stride duration (mean and SD), cadence (mean and SD), and sway (mean and SD). Recording days where walking was extracted relative to study begin are indicated by markers, dashed lines indicate recovery trends.

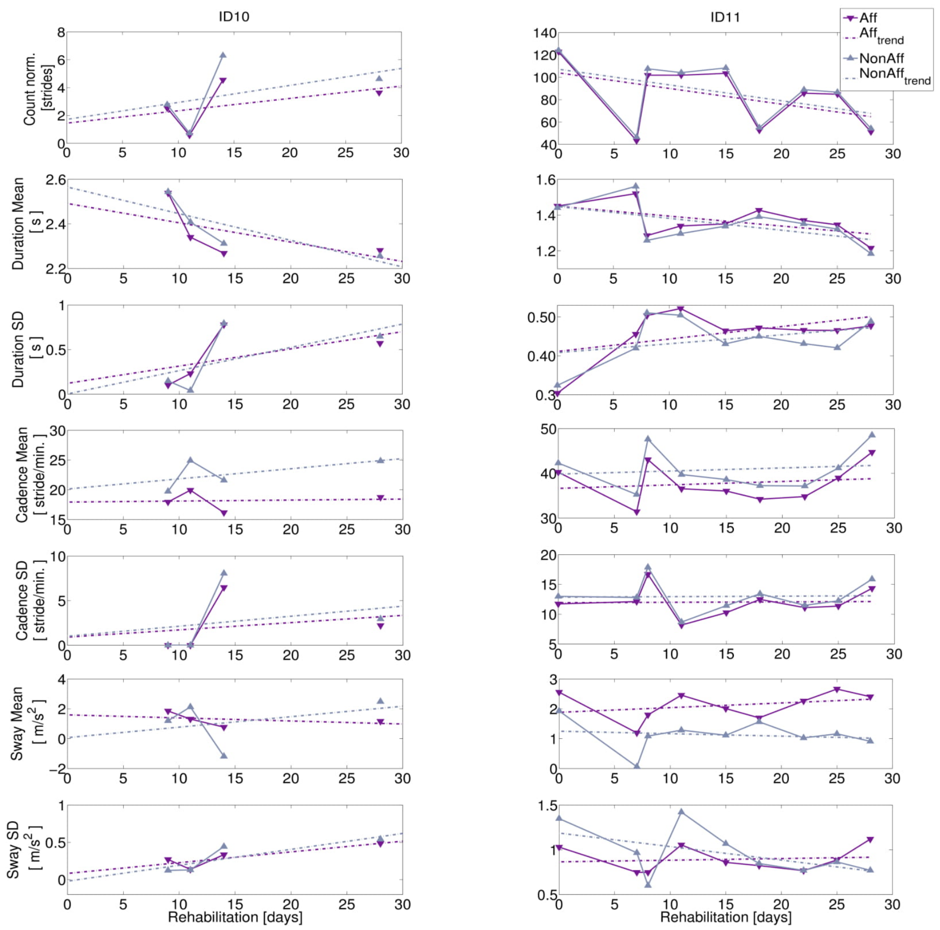

Figure S3. Bilateral trend analysis. Left column: wheelchair user (ID10, age = 68 years, 9 recording days), right column: walker (ID11, age = 55 years, 9 recording days). Top to bottom: extracted movement parameters: normalised stride count, stride duration (mean and SD), cadence (mean and SD), and sway (mean and SD). Recording days where walking was extracted relative to study begin are indicated by markers, dashed lines indicate recovery trends.
